# Rapid and Accurate Prediction and Scoring of Water Molecules in Protein Binding Sites

**DOI:** 10.1371/journal.pone.0032036

**Published:** 2012-03-01

**Authors:** Gregory A. Ross, Garrett M. Morris, Philip C. Biggin

**Affiliations:** 1 Structural Bioinformatics and Computational Biochemistry, University of Oxford, Oxford, United Kingdom; 2 InhibOx, Oxford, United Kingdom; Semmelweis University, Hungary

## Abstract

Water plays a critical role in ligand-protein interactions. However, it is still challenging to predict accurately not only where water molecules prefer to bind, but also which of those water molecules might be displaceable. The latter is often seen as a route to optimizing affinity of potential drug candidates. Using a protocol we call WaterDock, we show that the freely available AutoDock Vina tool can be used to predict accurately the binding sites of water molecules. WaterDock was validated using data from X-ray crystallography, neutron diffraction and molecular dynamics simulations and correctly predicted 97% of the water molecules in the test set. In addition, we combined data-mining, heuristic and machine learning techniques to develop probabilistic water molecule classifiers. When applied to WaterDock predictions in the Astex Diverse Set of protein ligand complexes, we could identify whether a water molecule was conserved or displaced to an accuracy of 75%. A second model predicted whether water molecules were displaced by polar groups or by non-polar groups to an accuracy of 80%. These results should prove useful for anyone wishing to undertake rational design of new compounds where the displacement of water molecules is being considered as a route to improved affinity.

## Introduction

Water is a key structural feature of protein-ligand complexes and can form a complex hydrogen-bonding network between ligand and protein [1], [2]. Water-mediated binding is so common that a study of 392 protein-ligand complexes found that 85% had at least one or more water molecules that bridge the interaction between the ligand and the protein [3]. Furthermore, the displacement of an ordered water molecule can drastically affect a ligand's binding affinity [4], [5]. As a result, it is common to include explicit water molecules in computational drug design [6]–[8]. The careful consideration of hydration sites has been shown to aid the predictability of 3D QSAR models, [9]–[11] ensure stable simulations with molecular dynamics [12], and improve the accuracy of rigorous free energy calculations [13]. Continuum solvent models have also been reported to improve with the addition of explicit water molecules [14]. Traditionally, ordered water molecules were ignored in ligand docking studies and ligands were docked into desolvated binding sites. There are now a number of docking protocols that include explicit water molecules and claim to improve accuracy in many cases [15]–[20]. However, it has also been reported that including such water molecules may hamper efforts to predict a ligand's correct binding mode [21].

A popular strategy in rational drug design is to modify a ligand so that it displaces an ordered water molecule into the bulk solvent [5], [11], [22], [23]. This is due to the favorable entropic gain that can result by increasing the water molecule's translational and orientational degrees of freedom. However, the targeted displacement of an ordered water molecule may be unsuccessful [24], [25], can also lead to a decrease in affinity if the ligand is unable to replace the water molecule's hydrogen bonds correctly and fulfill its stabilizing role [4], [26]. This has important implications for lead-optimization and rigorous theoretical studies have investigated how changing a water displacing functional group affects a ligand's affinity [27], [28]. In addition, water molecules are important pharmacophoric features of a binding site [29], and the chemical diversity of potential inhibitors generated *in silico* has been reported to be greatly affected by the targeted displacement of ordered water molecules [30]–[32]. Water molecule locations are typically taken from X-ray crystal structures and may be validated by observing the same position in other crystal structures of the same protein. Nevertheless, there are inherent problems with identifying hydration sites with crystallography. Water molecules can be artifactual, may be too mobile to identify or not observed because of low resolution [33]–[35]. In cases such as homology modeling, there will be no structural knowledge of water molecules. Hence, it is necessary to be able to accurately predict water locations within binding sites.

Water sites can be predicted by running molecular dynamics or Monte Carlo simulations with an explicit water model and taking the peaks in water density or averaging over water molecule locations [36]. These techniques have the benefit of including entropic effects in the prediction but can be very time consuming to run, especially with buried cavities due to the long time it takes for water to permeate within the protein. Grand canonical Monte Carlo methods can significantly reduce the length of the simulation [37], although can still be computationally demanding. The grid-based Monte Carlo method JAWS attempts to strike a balance between rapid solvation techniques and full molecular simulations that explicitly treat entropic effects [28]. It has the added advantage of producing an estimate of the free energy of displacing the water molecule into bulk solvent although the value may not be well converged [38]. A notable integral theory approach, called the 3D reference interaction site model (3D-RISM), has reported success in predicting the solvation structure within protein cavities [39] and in ligand binding sites [40]. Inhomogeneous fluid solvation theory (IFST), as popularized by Lazaridis [41], [42], uses a short molecular simulation to calculate the thermodynamics of water molecules in protein binding sites. A great advantage of using IFST is that the free energy is broken down into its enthalpic and entropic contributions and these values are then used to understand the thermodynamics of ligand binding [43]–[46]. IFST also forms the basis behind WaterMap [47], [48], which calculates the binding thermodynamics of displaced water molecules and has been used to understand the affinity and ligand selectivity in a number of different cases [49], [50].

Fast solvation methods have also been pursued for a number of years. A popular empirical method is GRID, which calculates the interaction energy of a chemical probe around a protein [51]. The water probe is able to make up to 4 hydrogen bonds with the protein. A novel mean field method has been reported by Setny and Zacharias that places potential water sites on a lattice and iteratively solves the solvent distribution using a semi-heuristic cellular automata approach [52]. The fact that water sites form distinctive distributions around amino acids [53] has been exploited by a number of knowledge-based methods. An early example called AQUARIUS predicted solvent sites within a protein by mapping each amino acid to a data set of crystal structures [54]. SuperStar is another knowledge-based method that combines structural data from the Protein Data Bank [55] and the Cambridge Structural Database [56] (CSD) to predict chemical propensity maps within protein cavities [57]. Schymkowitz et al. similarly used water distributions around amino acids to predict buried water molecules [58]. The distributions were clustered and then optimized using the Fold X forcefield. When water molecules that were coordinated by 2 or more polar atoms were considered, Fold X reported a success rate of 76%. Most recently, Rossato et al. developed AcquaAlta, which identified favorable water geometries from the CSD and *ab intio* calculations to predict the location of water molecules that bridge polar interactions between the ligand and the protein [59]. AcquaAlta predicted 76% of crystallographic water positions in the training set and 66% in the test set.

As the affinities, binding modes and chemical diversity of a series of ligands can be greatly affected by the water molecules in a protein binding site, it is important to predict which water molecules are displaced or conserved during the binding process. Some docking procedures, although different in implementation, involve switching explicit water molecules “on” and “off” [17], [60], [61]. Other approaches have used the structural features of a water molecule's environment to predict whether it will be displaced or not without any prior knowledge of the ligand. Using a K-nearest neighbors genetic algorithm, Consolv reported 75% accuracy in predicting whether a binding site water molecule would be displaced or not [62]. However, as Consolv used crystallographic temperature factors as structural descriptors, it cannot be applied to predicted water sites. Amadasi and co-workers have combined the HINT forcefield [63] with the Rank score [64] to classify water molecules into 2 broad categories; conserved/functionally displaced and sterically displaced/missing [65], [66]. Their first study correctly classified 76% of the water molecules tested while their second study reported a classification accuracy of 87%. Their analysis included weakly bound water molecules, which were a maximum of 4 Å away from the protein. On the other hand, WaterScore used water molecules within 7 Å of the bound ligand in protein-ligand binding sites [67]. Using multivariate logistic statistical regression, WaterScore reported 67% accuracy in classifying displaced and conserved waters, although water molecules that were displaced because of steric clashes with the ligand were not included in their analysis. Barillari et al. used the computationally expensive double-decoupling method to calculate the binding energies of 54 water molecules in protein-ligand complexes [68]. They found that water molecules that could be displaced by a ligand were on average less strongly bound than conserved water molecules by 2.5 kcal/mol.

Despite the positive strides that have been made in understanding the role of ordered waters, no single method is able to answer how displaceable a water molecule is, and what is it likely to be displaced by. When there is limited experimental knowledge of a binding site's solvation structure, addressing these questions becomes even less clear. In this paper we develop a pipeline that can accurately predict the location of water molecules and predict whether they are likely to be conserved or displaced after ligand binding. We also predict the probability that predicted water molecules will be displaced by polar or non-polar groups.

Using a method we call WaterDock, we show that the freely available AutoDock Vina tool [69] can be used to predict the location of ordered water molecules in ligand binding sites to a very high degree of accuracy. Crucially, a WaterDock prediction only takes a matter of seconds to produce. WaterDock was validated against high-resolution crystal structures, neutron diffraction data and molecular dynamics simulations. Using a validation set of proteins for which high resolution X-ray structures have been determined at least twice, we found that WaterDock was able to predict 88% of “consensus” water sites with a mean error of 0.78 Å. Using 14 structures of OppA bound to lysine-X-lysine tripeptides, WaterDock predicted 97% of the ordered water molecules, with on average 1 false positive per structure.

By combining data mining, heuristic and machine learning techniques, we developed two probabilistic water molecule classifiers that were designed to predict the role of our WaterDock predictions. Water molecules were predicted in the binding sites of the Astex Diverse Set [70] of protein-ligand complexes after the ligands had been removed from the structures. By overlaying the ligands back into the hydrated cavities, we studied the statistics of hypothetically “displaced” water molecules. We could predict whether water molecules were displaced or conserved to an accuracy of 75% and whether water molecules were displaced by a polar ligand group or a non-polar group to 80% accuracy, both after cross validation.

The key advantages of the approaches we present here are that they take only a few seconds to compute yet are able to maintain a very high degree of accuracy. We hope that these techniques will be useful in molecular modeling and rational drug design, especially in cases where there is limited structural information of the protein. Furthermore, they utilize freely available software.

## Methods

### 1. Validation of WaterDock method

Docking is a multidimensional optimization problem so many programs should be well adapted at balancing the various energetic needs of a water molecule. The main benefit of using AutoDock Vina (henceforth referred to as Vina) to predict water locations is that the stochastic nature of its algorithm ensures that many possible water sites can be generated in a single docking run. Repeated independent dockings of a water molecule into a cavity produces a diverse ensemble of locations that must be processed in order to produce a single, coherent and reproducible solvation structure. To ensure the prediction method is as fast as possible (Vina only takes a few seconds to dock a water molecule), we chose to experiment with different energetic filtering and clustering procedures. We refer to the docking, filtering and clustering procedure as WaterDock. Other docking programs can in principle be used to predict hydration sites within proteins and can be validated using the methods outlined in this paper.

**Figure 1:**
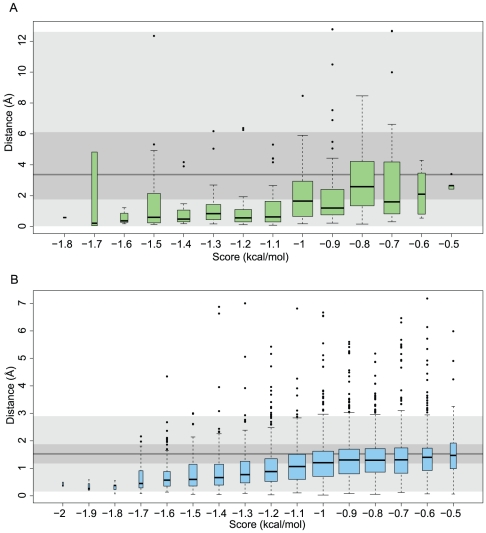
Box-plot summarizing the Vina score (in kcal/mol) versus the minimum distance (in Å) between the prediction and a crystallographic water (A) and MD water (B) from the data set in Table 1. Each box's lower and upper limits are at the 25% and 75% confidence limits. The solid black line within each box indicates the median. The width of each box is proportional to the square root of the number of data points. Outliers are shown as black dots and are defined by points outside 1.5 times the interquartile range. For comparison, the results from a random placement of water molecules are shown by the grey background box (light grey represents the whiskers, darker grey represents the 25% and 75% confidence limits and the darkest grey line represents the median). The accuracy of the placement increases with a more negative score and all predicted sites with scores less than −0.5 kcal/mol are better than random.

We used two data sets to validate WaterDock and one independent test set. The first validation set was used to find the minimum score for accepting a docked water site and the second validation set was created to establish the clustering procedure. By using 2 data sets to validate WaterDock, we hoped to minimize over-fitting the water placement method. The first set comprised of 15 high-resolution, pharmacologically relevant protein crystal structures and is shown in Table 1. As there can be some inconsistencies regarding crystallographically observed water molecules, it may be that Vina correctly predicts hydration sites that are not observed experimentally. For this reason, three proteins from Table 1 were chosen for molecular dynamics (MD) simulations. The minimum distances from predicted water molecules to an experimental or MD water molecule were used to investigate the relationship between a prediction's error and its Vina score. In order to assess the magnitude of the errors, the minimum distances were compared to those from a random placement of water molecules (see Figure 1). The energy cutoff was chosen as the Vina score that produced an error distribution that was indistinguishable from the error distribution from the random placement model.

**Table 1:** The protein structures used to establish a cut-off score that indicates whether or not a prediction is better than random.

Protein	PDB code	Resolution (Å)	Ligand
BRD4	2OSS	1.35	None
BRD4	3MXF	1.6	JQ1
Trypsin	1SOQ	1.02	None
Trypsin	1BTY	1.5	Benzamidine
HSP 90^a^	1AH6	1.8	None
HSP 90	1AM1	2	ADP
Penicillopepsin^a^	3APP	1.8	None
Penicillopepsin	1BXQ	1.41	PPi3
Cytochrome P450 2B4	1PO5	1.6	None
Cytochrome P450 2B4	1SUO	1.9	4-(4-chlorophenyl) imidazole
PIM1 kinase^a^	1YWV	2	None
PIM1 kinase	1XWS	1.8	BI1
Purine nucleoside phosphorylase	1V48	2.2	DFPP-G
GluA2 ligand binding core	1FTM	1.7	AMPA
HIV-1 protease	1KZK	1.09	JE-2147

a Structures that were selected for molecular dynamics simulations.

Table 1 includes *apo* and *holo* crystal structures of some of the same proteins in order to test whether Vina can predict the location of bridging water molecules as well as water molecules in unliganded binding sites. The proteins were also selected to have a diverse number of water molecules in the binding site. For example, trypsin has only one water molecule bridging the interaction between the ligand (benzamidine) and the protein whereas heat shock protein 90 has 9 bridging water molecules and 6 neighboring waters with its ligand, adenosine diphosphate (ADP). The unliganded structures of heat shock protein 90, penicillopepsin and PIM1 kinase were simulated using unrestrained MD for 10 ns. These proteins were selected as their binding sites vary in their hydrophobicity and are easily accessible to the bulk solvent. One hundred snap-shots were selected at random from the 3 simulations and Vina was used to predict the hydration sites in each snap-shot. Because of the hydrophobic diversity of the binding sites and a total of 300 conformational snap-shots were used for docking, we felt the number of simulations was sufficient to encapsulate different water structure in MD. Details of the MD simulations are provided in Text S1.

For each crystal structure or MD snapshot, Vina was used to dock a single water molecule into the binding site and all the locations and poses were recorded. The ensemble of different binding modes that are generated form the basis of the water site predictions. In a single run, Vina can generate a maximum of 20 conformations. Vina was used twice on each structure so there were 40 water site predictions for each binding site with overlap in many of the predicted positions. Using the Python [71] script that accompanies the software package AutoDockTools [72], the structures were stripped of water molecules and prepared into the appropriate PDBQT file format necessary for Vina. For *holo*-proteins, the search space was defined to be a 15 Å around the geometric center of the ligand. *Apo*-proteins were structurally aligned to the corresponding *holo* structure and the ligand center was again used to define the docking search space (See Text S1 for details).

As mentioned, Vina's predictions were compared to a random distribution of water molecules. Water molecules were placed at random within the sterically allowed volume of each docking search space. AutoGrid (part of the AutoDock 4 package) [73] was used to create oxygen affinity grid maps and favorable points were selected at random on grid locations that had affinities less than or equal to 0 kcal/mol. Five hundred random points were selected for each protein structure.

Repeated independent water molecule dockings creates many overlapping and similar water predictions even after low energy sites have been removed. A second data set was created in order to test the accuracy of different clustering methods and different docking procedures. An *accurate* water placement method is one in which many experimental water positions are correctly identified (high true positive rate) with very few predictions that are not experimentally observed (low false positive rate). As discussed in the introduction, the validity of water molecules seen in X-ray crystal structures is often uncertain and many water molecules may be missing from the structure. This complicates the proper assessment of the sensitivity and specificity of a water placement method.

To circumvent these issues, the data set in Table 2 was assembled in which each structure had been determined to a high resolution more than once. Where possible, neutron diffraction data was included because of its ability to resolve proton positions. Each protein in Table 2 was structurally aligned and “consensus” water molecules were determined. A consensus water molecule was defined as one that was within 1 Å of another water molecule seen in at least one other structure. These water molecules were used to assess the true positive rate of WaterDock. The binding site water molecules that were seen in only one structure were retained in order to quantify the false positive rate of WaterDock. By validating WaterDock in this way, WaterDock's true positive rate was assessed using only trustworthy water sites while its false positive rate was assessed using all water sites, for which there is at least some evidence for. Note that because of the difficulty in experimentally resolving some water molecules, the false positive rate is likely to be an upper estimate.

**Table 2:** The proteins and set of structures used to establish the docking and clustering procedures for the water placement method.

Protein	PDB codes	Resolution (Å)	Ligand
HIV-Protease	3FX5, 1HPX, 2ZYE^a^	0.93, 2,1.9	KNI-272
Ribonuclease A	1KF5, 1FS3, 5RSA^a^	1.2, 1.4, 2	None
GluR2 ligand binding core	1FTM^b^, 1MY2^b^	1.7, 1.8	AMPA
Trypsin	1S0Q, 1UTQ, 1TPO	1.0, 1.2, 1.7	None
Concanavalin A	1NLS, 1GKB, 1JBC, 1QNY^a^	0.9, 1.6, 1.2, 1.8	None
Glutathione S-transferase	1K3Y^b^, 1K3L^b^	1.3, 1,5	S-hexyl glutatione
Carbonic Anhydrase	3KS3, 3MWO, 2ILI	0.9, 1.4, 1.1	None

a Structures that have been determined by neutron diffraction.

b Structures where multiple chains have been used to validate ordered water molecules.

Each of the proteins in Table 2 were structurally aligned and consensus water sites were identified using the statistical programming language R [74]. Using a 15 Å cube to define each binding site, 185 distinct water molecules were identified. Of these water molecules, only 92 had been identified by at least twice by experiment. Observing less than half of experimentally determined water molecules in at least two structures highlights the uncertainty regarding crystallographic water positions and underlies the need for caution when validating a water prediction method.

To test WaterDock on an independent data set, we chose 14 structures of OppA bound to different KXK tri-peptides (see Table S1 and S2). The data set was primarily chosen because the same test set was used for a recent water prediction method called AcquaAlta [59]. Doing so allows a direct comparison of the two methods. In addition, the structures have been determined to a high resolution and the ligands have varied water distributions around the side chain of the central amino acid [2].

### 2. Investigating water displacement and conservation

When a ligand binds to a protein, water molecules that once occupied the ligand's position can be moved or displaced into the bulk solvent. As discussed in the introduction, the displacement of certain water molecules can have a profound effect on the affinity of a ligand. Hence, for each WaterDock prediction, we created a model to assign the probability that it will be either displaced or conserved during ligand binding. Such a probability effectively acts as a physically meaningful “score” that would help to identify which water sites are structurally important. We developed probabilistic models rather than discrete classifiers because whether a water molecule is displaced or not depends on the size, type and scaffold of a ligand. Classifying a water molecule as either *always* displaceable or *only* conserved we felt was an oversimplification.

As described in more detail below, we established three structural descriptors of water molecules in a binding site. Using a data mining protocol outlined below, we found a descriptor that correlates with the binding energy of a water molecule as calculated by thermodynamic integration. The two other descriptors were designed heuristically to encapsulate the hydrophilicity and lipophilicity of a water molecule's protein environment. As we wanted our probabilistic classifier to apply to our WaterDock predictions, we predicted water sites in a high quality data set of protein ligand complexes after the ligands had been removed from the structures. By overlaying the ligands back into the WaterDock solvated cavities and comparing the predicted water sites to crystallographic water molecules, we marked WaterDock predictions as either conserved or displaced. The hypothetically displaced water molecules were also recorded as being displaced by hydrogen-bonding groups or non-polar ligand groups. This approach allowed us to create a classifier that was consistent with our water placement method and circumvented issues relating to the displacement of water by protein side chain movements. Also, since WaterDock was found to be very accurate (see Results and Discussion), we were confident in our predictions of “*apo”* hydration sites.

Using a tree-based machine-learning algorithm, we created two models. The first assigned the probability that a water molecule will be either displaced or conserved. The second model assigned the probability that a water molecule will be displaced by a hydrogen-bonding group or a non-polar group.

#### Establishing a water energy score

Using the double decoupling method, Barillari et al. calculated the absolute binding free energies of 54 water molecules from 35 ligand-protein complexes [68]. The data set was made up of 6 proteins and 11 conserved water molecules. They found that conserved water molecules had statistically significant lower binding energies than displaceable water molecules. We considered this data set to be ideal to find the water energy score because of the size of the set, the diverse range of proteins and the consistent manner in which the binding energies were calculated. Each of the 54 water molecules were initially scored using the scoring functions from Vina and AutoDock 4 and correlations with R^2^ values of 0.01 and 0.31 were found. We felt these correlations were not strong enough to capture the calculated water energetics so we used a combination of AutoDock 4's force-field based scoring function and Vina's empirical scoring function as the starting point for a data mining procedure to find a new water energy model. All unique combinations of the terms in AutoDock 4 and the AutoDock Vina scoring functions were combined and fitted to Barillari's calculated binding data, creating 255 linear models. The models omitted terms relating to rotatable bonds, as they are not applicable to a water molecule. In order to avoid over-fitting, to reward model simplicity and hence find the most “meaningful” combination of terms, the models were then ranked by their Akaike information criterion (AIC) [75]. The AIC is a measure of the goodness of fit that penalizes models for the number of parameters they contain. The preferred model being the one that minimizes the AIC. The top 30 models with the lowest AICs were then selected for an extensive cross validation study.

To cross-validate the models, all the calculated binding data for one of the 11 conserved water molecules was partitioned from the training set to form a test set. The top 30 models were then re-fit to the training set and the mean error of the model on the test set was recorded. The process was repeated until each of the 11 conserved water molecules was used as the test set. The model that had the lowest mean error after cross-validation was selected as the final water energy model.

#### Creating heuristic hydrophilic and lipophilic scores

By analyzing 10,837 surface bound water molecules in 56 high resolution crystal structures, Kuhn et al. established the individual hydration propensities for each amino acid atom type [76]. They determined the propensities by dividing the total number of water molecules that hydrated an atom by the number of surface exposed occurrences. Building on their work, we created a hydrophilicity model and a lipophilicity model intended to encapsulate the local chemical environment of a water molecule. This information was intended to be distinct from the water energy model. The hydrophilicity model is a distance weighted sum of the propensities from all the atoms within 4 Å of a water molecule and is given by:
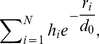
(1)where *N* is the number of protein atoms within 4 Å of the atomic position, *r_i_* is the distance (in Angstroms) of atom *i* to a water molecule, *h_i_* is the hydration propensity of atom *i* and *d_0_* is the distance scale of the interaction, set at 1 Å. We chose the weighting function because previous work have suggested that hydrophobicity decays exponentially with distance [77]. The hydration propensities of cofactor atoms were assigned the same value as the most similar protein atom. Because of the high magnitude of ion hydration free energies, ion hydration propensities were assigned the same as the highest value in the Kuhn data set. For the lipophilic score, we chose the same form as (1) and it is given by
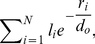
(2)where the terms are as before except *l_i_* which is the carbon propensity of atom *i*. As atomic carbon propensities have not been established as they have been for hydrophilicity, as a working hypothesis, we set all carbon atoms a propensity score of 1 and all other atom types a score of 0.

#### Finding displaced water molecules retrospectively with WaterDock

The Astex Diverse Set contains 85 high-resolution crystal structures of pharmacologically relevant ligand-protein complexes [70]. The ligands are drug-like and have a diverse range of scaffolds. Importantly, the electron density of the ligands in the crystal structures accounts for all parts of the ligand, leaving little ambiguity over the binding mode. This makes the Astex Diverse Set an appropriate data set to investigate what types of ligand atoms “displace” the WaterDock predictions.

The protein-ligand complexes were prepared for docking as previously described in this article. Ligands and water molecules were removed from the binding sites and cofactors were retained. Water sites were predicted in the binding site using the WaterDock method. A predicted water molecule was classified as conserved if it was seen within 1.5 Å of a water molecule seen in the crystal structure of the protein-ligand complex. Predicted water molecules that were not within 1.5 Å of a crystallographic water molecule but within 1.5 Å of a ligand atom were classified as displaced. The distance cut off was chosen as this represents an acceptable water prediction error and is within the van der Waals radius of a water molecule [78].

#### Creating a probabilistic water classifier

We expected that the displacement probability of a water molecule depended on a non linear combination of the 3 structural descriptors (binding energy, hydrophilicity and lipophilicity) and that certain regions of parameter space would generally correspond to different classes of water molecule. Classification trees meet these requirements by recursively partitioning the parameter space such that each region defines a class. Classification trees are particularly well suited to our problem because the proportion of a class in a partitioned region can be readily interpreted as a conditional probability. However, because of a tree's hierarchical nature, small changes in the data can result in a different series of splits, making single classification trees unstable. The method of bootstrap aggregation (known as “bagging”) alleviates this issue by fitting many trees to bootstrapped samples (sampling with replacement) of the data. The probability of a class is found by averaging the class proportions from each classification tree.

Using the free statistical language R with the package “rpart” [74], a bagged classification tree was written and was trained on the predicted water positions in Astex Diverse Set to classify them as conserved or displaced. In addition, a second model was trained to classify displaced WaterDock predictions as displaced by hydrogen-bonding groups or by non-polar groups. To assess the accuracy of the models, we used “leave-protein-out” cross validation. This involved partitioning the Astex Diverse Set into a training set and a test set, where the test set comprised of all the water molecules from a single protein. Each water molecule in the test set was classified by both models and the fraction of correct predictions were recorded. This process was repeated until all 85 proteins had been used as the test set. The accuracies quoted in the results are the mean accuracies from all the partitions. This validation procedure was chosen so that the models were tested on structures that were distinct to the structures in the training set.

## Results and Discussion

### 1. Validation of WaterDock as a Water Placement Tool

#### Determining the energetic cutoff

The minimum distance of each docked water molecule from a crystallographic or molecular dynamics (MD) water molecule was computed in order to assess how placement prediction error depended on the water position's Vina score. In particular, we sought to find a score cutoff that identified well-determined sites by comparing the predictions to a random placement of water molecules. Figure 1 shows how each Vina score has an error distribution associated with it and how the median and the range of the error distributions decreases for more negative scores. In particular, as the scores increase, the distributions tend to the error distribution from the random placement model. It is apparent that the lower the Vina score, the closer the agreement with crystallographic water locations.

When predicting water locations in the X-ray crystal structures of Table 1, the error distributions were always better than the error distribution from the random model. During the MD simulations, large numbers of water molecules filled the cavities. This meant that placing a water molecule at random within the cavity has a much greater chance of being near a simulated water molecule. While this meant that the prediction error was also reduced, improving on the random model provided a more stringent test. As a result, a cut-off of 0.6 kcal/mol was chosen by inspection as the minimum acceptable score of a predicted water molecule.

#### Establishing the docking and clustering method

Using 7 crystal structures that had been resolved multiple times (Table 2), different docking and clustering protocols were experimented with in order to find the method that predicted the largest number of consensus water molecules for the fewest number of false positives. Here, we summarize the most accurate protocol while the results for different docking and clustering regimes are included in Table S3.

We found that independently docking a water molecule 3 times into the binding site was enough to sufficiently sample the configuration space of the water molecule while docking only once did not. The “exhaustiveness” parameter in Vina determines how rigorous the docking search is and is roughly proportional to elapsed docking time. We found that setting this parameter to 20 significantly improved the accuracy of the subsequent clustering methods when compared to an exhaustiveness value of 10. Three independent docking runs with an exhaustiveness value of 20 was also very fast and took no more than 15 seconds to complete on a 2.33 GHz Intel Xeon quad core processor.

Independently docking a water molecule 3 times with Vina generates a maximum of 60 binding modes. Many of the positions overlapped or were in close proximity to one another. Clustering the water positions is a time efficient way of producing a solvation map of the binding site from an ensemble of water positions. A number of different hierarchical clustering methods were experimented with, including complete linkage, single linkage and Ward's minimum variance method. Distance cutoffs of each clustering method were varied to find the one that gave the best accuracy. The average position of a docked water molecule cluster was used as the predicted water molecule location.

The most accurate clustering method was found to be with 2 rounds of single linkage clustering with different distance cutoffs. The results are summarized in Tables 3 and 4. The first clustering round used a distance cutoff of 0.5 Å and was designed to remove the most overlapping sites and to reduce the “chaining” of clusters in the second docking round. The output was clustered again with a distance cutoff of 1.6 Å. While these distance cutoffs were established empirically so as to maximize accuracy, it is interesting to note that the second clustering cutoff is around the van der Waals radius of a water molecule [78].

**Table 3:** The performance of the final WaterDock method on the second validation set.

	Max Error = 1.5 Å			Max Error = 2.0 Å		
Maximum distance of experimental waters from protein (Å)	Consensus water molecules predicted (%)	False Positives (%)	Mean Error (Å)	Consensus water molecules predicted (%)	False Positives (%)	Mean Error (Å)
3	88	24	0.69	94	16	0.77
3.3	81	24	0.69	88	16	0.78

**Table 4:** The individual protein results using the final WaterDock method.

	HIV Protease	Ribonuclease A	GluR2	Trypsin	Concanavalin A	GST‡	Carbonic Anhydrase	Total
Consensus Waters	9	10	15	14	17	13	15	93
Predicted Consensus Waters	9	8	15	13	13	12	12	82
False Positives	2	3	3	2	4	3	4	21
Water Molecules Predicted*	18	20	20	17	21	19	18	133

* The number of correctly predicted non-consensus water sites can be calculated by finding the difference between the number of water molecules predicted and the sum of the predicted consensus waters and false positives.

‡ Glutathione S-transferase.

Using a maximum placement error of 2 Å the final WaterDock method identified 88% of consensus water molecules within 3.3 Å of the protein. The distance of 3.3 Å was chosen from the water-water radial distribution function so as to define the first hydration shell [79]. Out of the 80 consensus water molecules correctly identified, only 8 were over 1.5 Å away from the experimental position and 54 were within 1 Å of a consensus water molecule. When only tightly bound water molecules (within 3 Å of the protein) were considered, WaterDock predicted 94% of these consensus water molecules.

**Figure 2:**
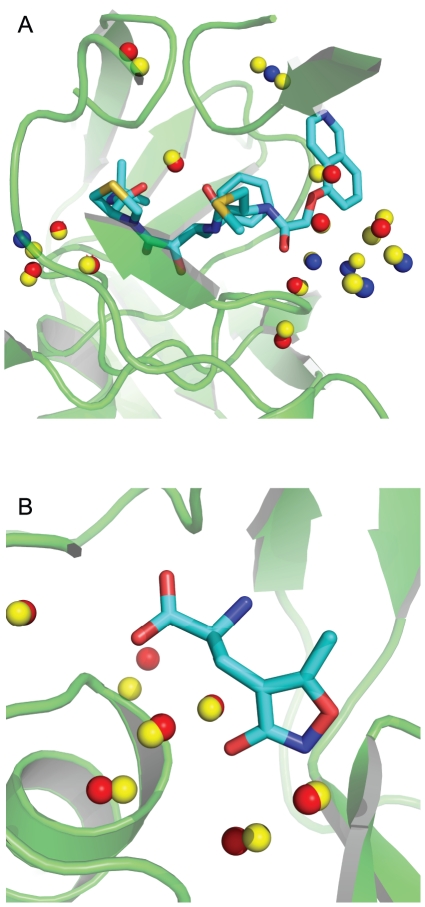
Two examples from the data set used to validate the WaterDock method. Yellow spheres: predicted water sites, red spheres: water molecules observed in at least two experimental structures, blue spheres: water molecules observed in only one experimental structure. HIV-1 protease bound to the inhibitor KNI-272 (A). All 9 consensus water molecules and all 6 non-consensus water molecules are correctly identified. One non-consensus water molecule is in between two predictions, resulting in a false positive. This water molecule was resolved only in 3FX5 with a temperature factor of 42 Å^2^, so the over-prediction may be due to the uncertainty in the water molecule's position. GluR2 ligand binding core bound to AMPA (B). All water molecules within the binding site are correctly predicted.

Given that only protein-water interactions and not water-water interactions were used to generate the initial ensemble of positions, it is perhaps surprising that WaterDock was able to predict the vast majority of consensus water sites. Even in examples that contain a complex network of water molecules, such as Ribonuclease A, and Carbonic Anhydrase, WaterDock was still able to predict 80% of the consensus sites (see Table 3). It is clear therefore, that the protein is the most important factor in determining a water molecule's position. However, the omission of water-water interactions was likely to be responsible for some of the errors. In a few cases, an experimental water site was found to lie between 2 predicted locations (see Figure 2), resulting in a false positive. In examples such as Ribonuclease A, Concanavalin A and Carbonic Anhydrase, it was found that water-water interactions were very subtle and consensus sites were observed to be slightly displaced with respect to the WaterDock predictions, possibly to accommodate and interact with another water molecule.

Water-water interactions could be included in the WaterDock method if a second sampling procedure, akin to the JAWS method [28] could switch the predicted sites “on” and “off”. We also considered sequentially docking a water molecule into a cavity to account for water-water interactions. However we found that the point at which to stop docking was ambiguous and that subsequent predictions were biased to regions near previous predictions. Importantly, neither of these methods adapt the positions of water molecules to optimize both the protein-water and the water-water interactions. A second energy minimization step would be required to achieve this. Given the high accuracy and speed of the current method, we felt these improvements were unnecessary. Table 4 shows the number of correctly predicted consensus water molecules and the number of mis-predictions for each individual protein.

#### Applying WaterDock to the test set

**Figure 3:**
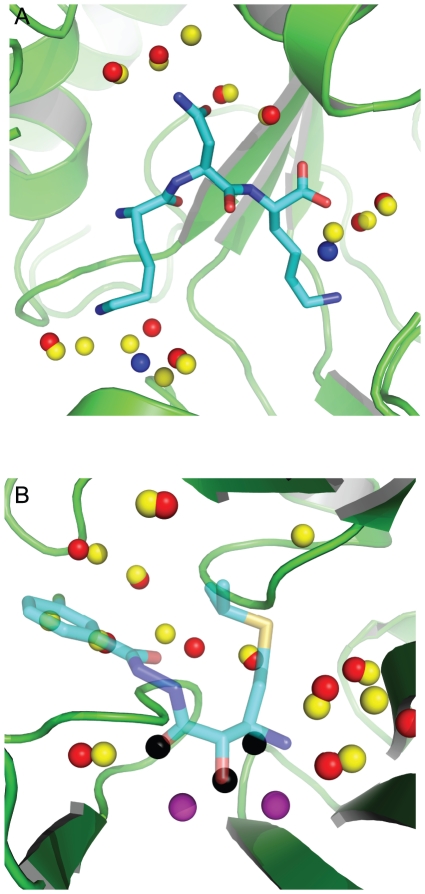
An example from the test set used to validate WaterDock. OppA is shown bound to the tripeptide KNK, PDB code 1B5I as shown in (A). Red spheres: crystallographic water molecules; blue spheres: water molecules seen in other related structures; yellow spheres: WaterDock predictions. All water molecules are correctly predicted with 2 false positives. An example from the retrospective displacement study: human methionine aminopeptidase-2 bound to an inhibitor (blue transparent sticks), PDB code 1R58 as shown in (B). Yellow spheres: water sites predicted in the absence of the ligand; black spheres: predicted water sites that overlap with the ligand; red spheres: crystallographic water molecules seen in the protein-ligand complex, purple spheres: manganese ions. Predictions that correspond to water molecules seen in the crystal structure are considered to be “conserved” and water molecules that overlap with the ligand are considered to be “displaced”. Three predicted water molecules are observed to be displaced by 2 oxygen and 1 nitrogen ligand atoms.

We decided to use to same data set used by the water prediction method, AcquaAlta [59], as our test set so as to allow a direct comparison of the methods. The test set comprised of fourteen crystal structures of OppA bound to different KXK tri-peptides. AcquaAlta reported that they could predict 66% of the water molecules that bridged the interaction between the ligand and the protein to a maximum error of 1.4 Å. Using the same maximum error, WaterDock predicted 87% of the crystallographic water molecules. When the results were visually inspected (Figure 3), 11 additional predictions were found to be within 2.0 Å of crystallographic water molecules that made the same interactions with the ligand and protein. When these water molecules were included in the analysis, WaterDock identified 97% of the crystallographic water sites with a mean error of 0.68 Å. On average, WaterDock predicted just under 1 water molecule per structure that was not seen experimentally. The false positive rate was not reported for AcquaAlta.

### 2. Predicting displaceable water molecules using WaterDock

#### Water energy model from a data mining procedure

The 54 water molecules that Barillari's et al. calculated the binding energy for using the double decoupling method [68] were scored with the AutoDock 4 and the Vina scoring functions. All linear combinations of the scoring functions energetic terms were used to create 255 energy models. After selecting the top 30 models based on model simplicity and goodness of fit (as denoted by the model's AIC), cross validation was used to find the model that yielded the lowest error. It was found that a single term, the hydrogen bonding term from Vina's scoring function had the lowest mean error in the cross-validation (CV) study, with an error of 1.7 kcal/mol. The standard error of the fit was 1.6 kcal/mol and had an R^2^ value of 0.50. For comparison, if the average calculated energy of the Barillari data set is used to predict each water molecule's energy, the mean error would be 2.5 kcal/mol. The coefficient and intercept of the re-weighted Vina hydrogen bonding term is shown in Table 5.

**Table 5:** The gradient and intercept of the Vina's hydrogen-bonding term after refitting it to the calculated binding energy of water according to Barillari et al.

Term	Weight (kcal/mol)
Intercept	1.77
H-bond	−2.58

Vina's hydrogen bonding term is the sum over hydrogen bonding pairs [69]. For each pair, the value ranges from 1 to 0 and varies linearly with distance. The significant correlation despite the simplicity of the model result is likely to be due to a strong enthalpy-entropy compensation effect, where the number and strength of hydrogen bonds correlates with the translational and orientational freedom of the water molecule.

#### Classifying the role of water

As displaced water molecules can greatly affect a ligand's affinity and specificity, it is of great interest to quantify the probability that a WaterDock prediction will be displaced or conserved. If a water is displaceable, it useful to know whether is likely to be displaced by a polar group or a non-polar group. In order to develop a water classifier that is consistent with our water placement method, we used a high quality data set of protein ligand complexes to predict the locations of water molecules after the ligands had been removed from the structures. By overlaying the ligands back onto the hypothetical *“apo”* solvation structure, we investigated the displacement statistics of our water predictions (See Figure 2B). In total, 545 predicted *apo* water molecules were within 1.5 Å of a water molecule seen in the crystal structure of the protein-ligand complex and so were classified as conserved. Also, 459 predicted water molecules were classified as displaced as they were within 1.5 Å from a ligand. Of these displaced water molecules, 216 were displaced by polar groups and 243 were displaced by non polar groups.

Using the re-weighted Vina hydrogen bond term, the hydrophilicity model and the lipophilicity model as descriptors in a probabilistic machine learning classifier, water molecules were predicted to be either being displaced or conserved. Using “leave-protein-out” cross validation (as described in Methods), 75% of the WaterDock predictions were correctly classified as either conserved of displaced when the class with the highest probability was used for the prediction. Similarly, when waters predicted to be displaced by WaterDock were classified as being displaced by a polar group or by a non-polar group, 80% of the WaterDock predictions were correctly classified in cross validation. Table 6 shows that there was little bias in predicting each individual class.

**Table 6:** The results of the models that classify water molecules as displaced or conserved and as displaced by a polar group and displaced by a non-polar group.

Model 1 correctly classified (%)			Model 2 correctly classified (%)		
Total	Conserved waters	Displaced waters	Total	Waters displaced by polar groups	Waters displaced by non-polar groups
75	70	81	80	82	79

One benefit of using a probabilistic classifier is that the certainty of a prediction is naturally quantified. One would therefore expect that the higher the classification probability is, the lower the chance of misclassification. For both of our models, we found that classification probabilities of 0.8 or above correctly classified the water in 94% and 95% of cases in both models after cross validation. This emphasizes the usefulness of the probabilistic approach taken.

**Figure 4:**
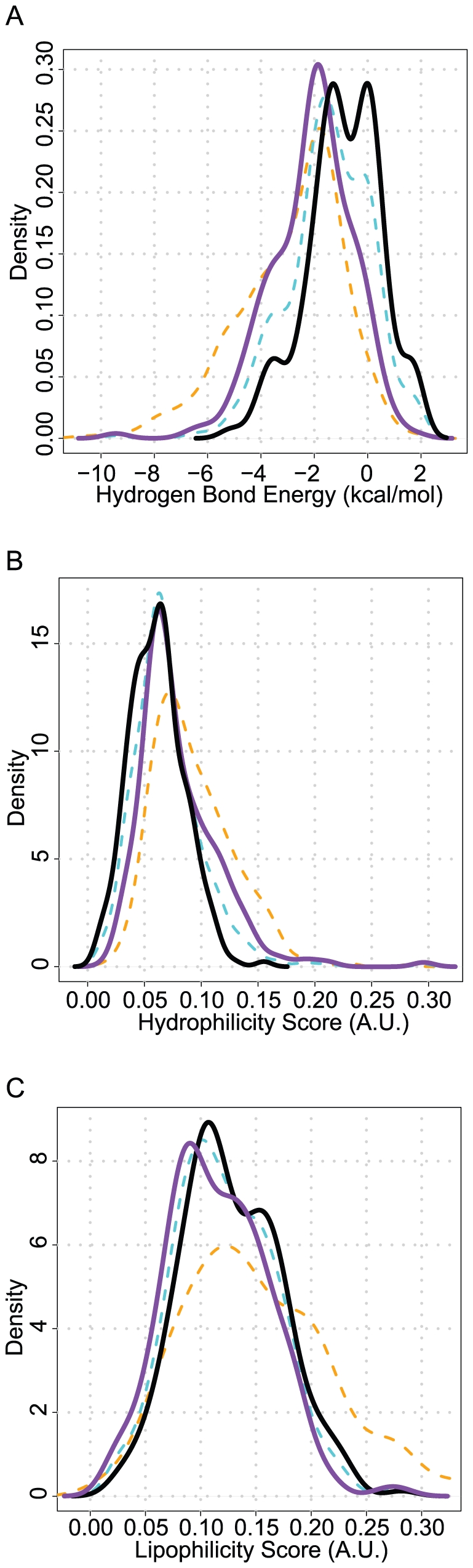
The distribution of energies of water molecules displaced by polar groups (purple) and by non-polar atoms (black) for the hydrogen bond score (A), hydrophilicity score (B) and the lipophilicity score (C). Overlaid are the distributions of energies of conserved water molecules (dashed orange) and displaced water molecules (dashed cyan). While each score can be used individually to classify water molecules, to obtain the accuracies shown in Table 6, all scores must be included in the machine learning classifier.

Figure 4 shows the distributions of the three scores for WaterDock predictions displaced by polar and non polar groups as well as for conserved and displaced water molecules. While each score could be used individually to distinguish between water classes, we found that the highest accuracy in the cross validation could only be achieved using all three energy scores (Tables S4 and S5).

In Figure 4, it seems counter intuitive that conserved WaterDock predictions are more likely to have a higher lipophilic score than displaced water molecules. This is due to the fact that conserved water molecules tend to be more buried and so have more contacts with the protein, which also explains the higher hydrophilicity scores and the stronger hydrogen bonds. The opposite is true when one compares WaterDock predictions that were displaced by polar groups to water predictions that were displaced by non-polar groups. Water molecules displaced by non-polar groups tend to reside in slightly more lipophilic and less hydrophilic environments and tend to make fewer and weaker hydrogen bonds.

It is interesting to note that even though Vina's hydrogen-bonding term was established using a data mining protocol and the hydrophilicity score was designed heuristically, both scores were strongly correlated with an R^2^ of 0.72. These very different approaches have converged to describe a related property of water. Despite the high correlation, the combination of the two scores in the machine learning algorithm increased the classification accuracy by around 7% compared to when each term was fitted individually (see Table S4). Because the increase in accuracy is seen after cross-validation, it indicates that it is not a result of over-fitting and, that despite the high correlation, the terms sufficient are sufficiently distinct so as to improve the classification success rate.

#### Ligand water displacement propensities

As well as predicting the role that WaterDock predictions play in ligand binding, we also investigated the propensities for ligand chemical groups to occupy predicted water sites. Given the very good agreement with WaterDock's predictions and experimentally determined water sites, we expect these displacement statistics to be similar for water molecules seen in crystal structures.

**Figure 5:**
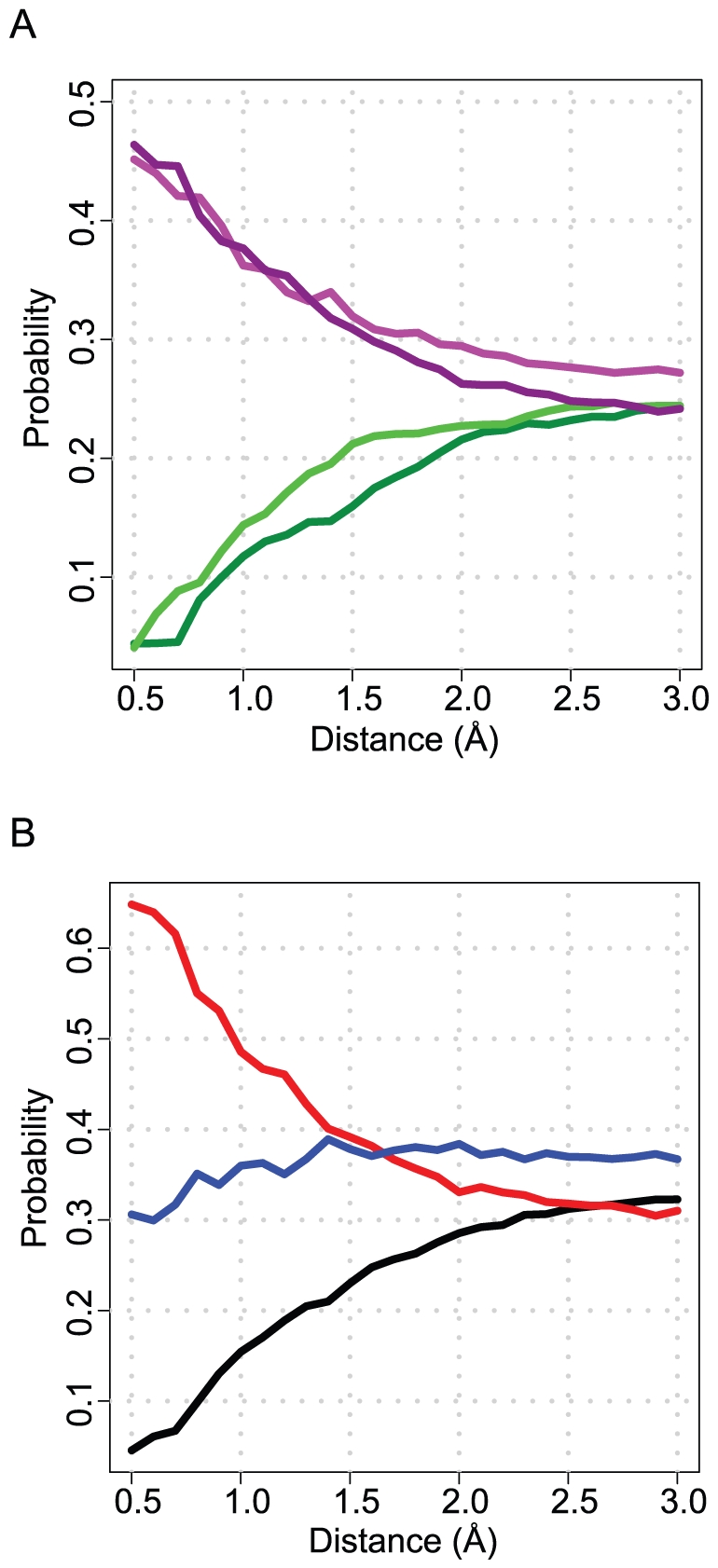
The probability of finding a ligand atom, by group, at a given distance from a predicted water site (A). Light purple: hydrogen donors, dark purple: hydrogen acceptors, light green: non-aromatic carbon atoms, dark green: aromatic carbon atoms. The probability of finding a ligand atomic element at a given distance from a predicted water site (B). Red: oxygen, blue: nitrogen, black: carbon. Within 1.5 Å, ligand oxygen atoms are far more likely to occupy predicted water sites than any other element.

Figure 5 shows the probability of finding ligand functional groups at various distances from hypothetically displaced water sites. For a given distance cutoff, each point can be considered as the propensity that a ligand atom will displace a water molecule. Hydrogen bond donors and acceptors were equally likely to displace predicted water molecules and were found to be around 9 times more likely to be within 0.5 Å of a water site than aromatic and aliphatic carbons. This indicates that it is important for water displacing ligand groups to replicate water's hydrogen bonding capacity. Interestingly, when the occupation probabilities were computed for ligand atoms, rather than atom functions, oxygen atoms were over twice as likely to be found within 0.5 Å of a displaced water site than nitrogen atoms. At 1.5 Å (the distance cutoff we previously used to define whether a water molecule was displaced or not) the displacement propensities of oxygen and nitrogen are roughly the same. The higher probability for a ligand oxygen atom to more closely occupy a displaced water site further emphasizes the importance for ligands groups to mimic the water molecule they displace.

As the distance from a predicted water site increases further, the less one can consider a ligand atom to have displaced a water molecule. As a result, the propensities tend to the same value. Ligand atoms such as halogens, sulfur and phosphorous were not included in this study due to their small number in the data set.

From Figure 5, it is tempting to conclude that ligand modifications designed to displace a water molecule should always be made with an hydrogen-bonding group. However, in this study we have seen that many water molecules, depending on their local environment, are preferentially displaced by non-polar groups. However, since carbon is the most abundant ligand element in the Astex Diverse Set and representative of drug-like ligands, the per atom displacement probability is significantly less for carbon than for polar atoms.

### Conclusions

Using three data sets, we have shown that by using a method we call WaterDock, the docking software AutoDock Vina can be used to predict the binding positions of water molecules in an accurate manner. Using structures that have been determined more than once by either X-ray crystallography or by neutron diffraction, we found WaterDock could predict 88% of consensus water molecules. In order to understand the structural importance of WaterDock's predictions, we combined data mining, heuristic and machine learning techniques to assess the probability that a prediction is either conserved or displaced. After cross-validation, this model had a classification accuracy of 75%. Similarly, we found we could predict whether WaterDock predictions were displaced by polar or non-polar ligand groups to an accuracy of 80%.

These models allow one to predict not only the location of water molecules, but also if a water is likely to be displaceable by oxygen or nitrogen atoms only or whether in fact there is scope for displacement by something more non-polar, like a methyl group. Such knowledge could be advantageous in the context of lead-optimization. Work is underway to see how this water scoring information can be used to improve the prediction of ligand-protein binding affinities. An example water-placement prediction script is available (Supporting Information S1) and all water classifiers are available on request.

## Supporting Information

Text S1 **Full details of the molecular dynamics procedure and the docking procedures used for specific proteins.**
(DOC)

Table S1 The X-ray crystal structures of OppA used as the test set for the water placement method. Fourteen crystal structures of OppA were used as the WaterDock test set. This test set was chosen to match the test set used by the water prediction method Acqua Alta. The water molecules used in our study are shown in Table S4. These water molecules bridge the interaction between OppA and the ligands. The listed water molecules were used to test the true positive rate of WaterDock. As all the ligands are lysine-X-lysine tripeptides, all the water molecules around the lysine residues were used to calculate WaterDock's false positive rate.
(DOC)

Table S2 Water molecules used in the OppA test set.
(DOC)

Table S3 Results of different water docking methods (performed on structures in Table 2 of the main manuscript). The final WaterDock method was chosen as the one that predicted the most number of consensus water molecules for the lowest false positive rate. Various docking parameters were experimented with as well as different clustering methods. To demonstrate how changing the docking or clustering parameters affects the prediction accuracy, some of the results of different water prediction methods are shown. The success rates shown are for a maximum error of 2 Å. The final method, shown in the bottom row of this table, was chosen after an exhaustive parameter and methods search.
(DOC)

Table S4 Classification accuracies for conserved and displaced waters. Three water scoring energy terms were established to describe a water molecules binding energy (AutoDock Vina's hydrogen bonding term) and the water molecules' local environment with our hydrophilic and hydrophobic terms. These scores were used in 2 bagged tree classifiers that predicted whether water molecules were displaced or conserved. The probabilistic classifiers were fit using all combinations of the water scores. Cross validation results are shown and demonstrate that all three scores must be included for maximum accuracy.
(DOC)

Table S5 Classification accuracies for waters displaced by polar and non-polar groups. The probabilistic classifiers were fit using all combinations of the water scores as for Table S5.
(DOC)

Supporting Information S1 A gzipped archived of the water-placement scripts used.
(ZIP)

## References

[1] Roe SM, Prodromou C, O'Brien R, Ladbury JE, Piper PW (1999). Structural basis for inhibition of the Hsp90 molecular chaperone by the antitumor antibiotics radicicol and geldanamycin.. J Med Chem.

[2] Sleigh SH, Seavers PR, Wilkinson AJ, Ladbury JE, Tame JR (1999). Crystallographic and calorimetric analysis of peptide binding to OppA protein.. J Mol Biol.

[3] Lu Y, Wang R, Yang C-Y, Wang S (2007). Analysis of ligand-bound water molecules in high resolution crystal structures of protein-ligand complexes.. J Chem Inf Model.

[4] Clarke C, Woods RJ, Gluska J, Cooper A, Nutley MA (2001). Involvement of water in carbohydrate-protein binding.. J Am Chem Soc.

[5] Lam PY, Jadhav PK, Eyermann CJ, Hodge CN, Ru Y (1994). Rational design of potent, bioavailable, nonpeptide cyclic ureas as HIV protease inhibitors.. Science.

[6] de Beer SB, Vermeulen NP, Oostenbrink C (2010). The role of water molecules in computational drug design.. Curr Top Med Chem.

[7] Mancera RL (2007). Molecular modelling of hydration in drug design.. Curr Opin Drug Discov Devel.

[8] Wong SE, Lightstone FC (2011). Accounting for water molecules in drug design.. Exp Opin Drug Discov.

[9] Hussain A, Melville J, Hirst J (2010). Molecular docking and QSAR of aplyronine A and analogues: potent inhibitors of actin.. J Comput Aided Mol Des.

[10] Pastor M, Cruciani G, Watson KA (1997). A strategy for the incorporation of water molecules present in a ligand binding site into a three-dimensional quantitative structure-activity relationship analysis.. J Med Chem.

[11] Taha MO, Habash M, Al-Hadidi Z, Al-Bakri A, Younis K (2011). Docking-based comparative intermolecular contacts analysis as new 3-D QSAR concept for validating docking studies and in silico screening: NMT and GP inhibitors as case studies.. J Chem Inf Model.

[12] Wallnoefer HG, Handschuh S, Liedl KR, Fox T (2010). Stabilizing of a globular protein by a highly complex water network: a molecular dynamics simulation study on factor Xa.. J Phys Chem B.

[13] Luccarelli J, Michel J, Tirado-Rives J, Jorgensen WL (2010). Effects of water placement on predictions of binding affinities for p38α MAP kinase inhibitors.. J Chem Theory Comput.

[14] Wallnoefer HG, Liedl KR, Fox T (2011). A challenging system: Free energy prediction for factor Xa.. J Comput Chem.

[15] de Graaf C, Oostenbrink C, Keizers PH, van der Wijst T, Jongejan A (2006). Catalytic site prediction and virtual screening of cytochrome P450 2D6 substrates by consideration of water and rescoring in automated docking.. J Med Chem.

[16] de Graaf C, Pospisil P, Pos W, Folkers G, Vermeulen NP (2005). Binding mode prediction of cytochrome P450 and thymidine kinase protein-ligand complexes by consideration of water and rescoring in automated docking.. J Med Chem.

[17] Rarey M, Kramer B, Lengauer T (1999). The particle concept: placing discrete water molecules during protein-ligand docking predictions.. Proteins.

[18] Roberts BC, Mancera RL (2008). Ligand-protein docking with water molecules.. J Chem Inf Model.

[19] Santos R, Hritz J, Oostenbrink C (2010). Role of water in molecular docking simulations of cytochrome P450 2D6.. J Chem Inf Model.

[20] Thilagavathi R, Mancera RL (2010). Ligand-protein cross-docking with water molecules.. J Chem Inf Model.

[21] Bellocchi D, Macchiarulo A, Costantino G, Pellicciari R (2005). Docking studies on PARP-1 inhibitors: insights into the role of a binding pocket water molecule.. Bioorg Med Chem.

[22] Chen JM, Xu SL, Wawrzak Z, Basarab GS, Jordan DB (1998). Structure-based design of potent inhibitors of scytalone dehydratase: displacement of a water molecule from the active site.. Biochemistry.

[23] Wissner A, Berger DM, Boschelli DH, Floyd MB, Greenberger LM (2000). 4-Anilino-6,7-dialkoxyquinoline-3-carbonitrile inhibitors of epidermal growth factor receptor kinase and their bioisosteric relationship to the 4-anilino-6,7-dialkoxyquinazoline inhibitors.. J Med Chem.

[24] Clarke C, Woods RJ, Gluska J, Cooper A, Nutley MA (2001). Involvement of water in carbohydrate-protein binding.. J Am Chem Soc.

[25] Kadirvelraj R, Foley BL, Dyekjaer JD, Woods RJ (2008). Involvement of water in carbohydrate-protein binding: Concanavalin A revisited.. J Am Chem Soc.

[26] Mikol V, Papageorgiou C, Borer X (1995). The role of water molecules in the structure-based design of (5-hydroxynorvaline)-2-cyclosporin: synthesis, biological activity, and crystallographic analysis with cyclophilin A.. J Med Chem.

[27] Garcia-Sosa AT, Mancera RL (2010). Free energy calculations of mutations involving a tightly bound water molecule and ligand substitutions in a ligand-protein complex.. Mol Inf.

[28] Michel J, Tirado-Rives J, Jorgensen WL (2009). Energetics of displacing water molecules from protein binding sites: Consequences for ligand optimization.. J Am Chem Soc.

[29] Lloyd DG, Garcia-Sosa AT, Alberts IL, Todorov NP, Mancera RL (2004). The effect of tightly bound water molecules on the structural interpretation of ligand-derived pharmacophore models.. J Comp Aided Mol Des.

[30] Garcia-Sosa AT, Firth-Clark S, Mancera RL (2005). Including Tightly-Bound Water Molecules in de Novo Drug Design. Exemplification through the in Silico Generation of Poly(ADP-ribose)polymerase Ligands.. J Chem Inf Model.

[31] Garcia-Sosa AT, Mancera RL (2006). The effect of a tightly bound water molecule on scaffold diversity in the computer-aided de novo ligand design of CDK2 inhibitors.. J Mol Mod.

[32] Mancera RL (2002). De novo ligand design with explicit water molecules: an application to bacterial neuraminidase.. J Comput Aided Mol Des.

[33] Carugo O, Bordo D (1999). How many water molecules can be detected by protein crystallography?. Acta Crystallogr D Biol Crystallogr.

[34] Davis AM, Teague SJ, Kleywegt GJ (2003). Application and limitations of X-ray crystallographic data in structure-based ligand and drug design.. Angew Chem Int Ed Engl.

[35] Ernst JA, Clubb RT, Zhou H-X, Gronenborn AM, Clore GM (1995). Demonstration of positionally disordered water within a protein hydrophobic cavity by NMR.. Science.

[36] Henchman RH, McCammon JA (2002). Extracting hydration sites around proteins from explicit water simulations.. J Comput Chem.

[37] Resat H, Mezei M (1996). Grand canonical ensemble Monte Carlo simulation of the dCpG/proflavine crystal hydrate.. Biophys J.

[38] Michel J, Essex JW (2010). Prediction of protein-ligand binding affinity by free energy simulations: assumptions, pitfalls and expectations.. J Comput Aided Mol Des.

[39] Imai T, Hiraoka R, Kovalenko A, Hirata F (2007). Locating missing water molecules in protein cavities by the three-dimensional reference interaction site model theory of molecular solvation.. Proteins: Struct Func Genet.

[40] Imai T, Oda K, Kovalenko A, Hirata F, Kidera A (2009). Ligand mapping on protein surfaces by the 3D-RISM theory: Toward computational fragment-based drug design.. J Am Chem Soc.

[41] Lazaridis T (1998). Inhomogeneous fluid approach to solvation thermodynamics. 1. Theory.. J Phys Chem B.

[42] Lazaridis T (1998). Inhomogeneous fluid approach to solvation thermodynamics. 2. Applications to simple fluids.. J Phys Chem B.

[43] Li Z, Lazaridis T (2003). Thermodynamic contributions of the ordered water molecule in HIV-1 protease.. J Am Chem Soc.

[44] Li Z, Lazaridis T (2005). Thermodynamics of buried water clusters at a protein at ligand binding interface.. J Phys Chem B.

[45] Li Z, Lazaridis T (2005). The effect of water displacement on binding thermodynamics: concanavalin A.. J Phys Chem B.

[46] Li Z, Lazaridis T (2006). Thermodynamics of buried water clusters at a protein-ligand binding interface.. J Phys Chem B.

[47] Abel R, Young T, Farid R, Berne BJ, Friesner RA (2008). Role of the active-site solvent in the thermodynamics of factor Xa ligand binding.. J Am Chem Soc.

[48] Young RJ, Campbell M, Borthwick AD, Brown D, Burns-Kurtis CL (2006). Structure- and property-based design of factor Xa inhibitors: pyrrolidin-2-ones with acyclic alanyl amides as P4 motifs.. Bioorg Med Chem Lett.

[49] Frydenvang K, Pickering DS, Greenwood JR, Krogsgaard-Larsen N, Brehm L (2010). Biostructural and pharmacological studies of bicyclic analogues of the 3-isoxazolol glutamate receptor agonist ibotenic acid.. J Med Chem.

[50] Robinson DD, Sherman W, Farid R (2010). Understanding kinase selectivity through energetic analysis of binding site waters.. ChemMedChem.

[51] Goodford PJ (1985). A computational procedure for determining energetically favorable binding sites on biologically important macromolecules.. J Med Chem.

[52] Setny P, Zacharias M (2010). Hydration in discrete water. A mean field, cellular automata based approach to calculating hydration free energies.. J Phys Chem B.

[53] Thanki N, Thornton JM, Goodfellow JM (1988). Distributions of water around amino acid residues in proteins.. J Mol Biol.

[54] Pitt WR, Goodfellow JM (1991). Modelling of solvent positions around polar groups in proteins.. Protein Eng.

[55] Berman HM, Westbrook J, Feng Z, Gilliland G, Bhat TN (2000). The Protein Data Bank.. Nucleic Acids Res.

[56] Allen FH (2002). The Cambridge Structural Database: a quarter of a million crystal structures and rising.. Acta Crystallogr B.

[57] Verdonk ML, Cole JC, Taylor R (1999). SuperStar: a knowledge-based approach for identifying interaction sites in proteins.. J Mol Biol.

[58] Schymkowitz JW, Rousseau F, Martins IC, Ferkinghoff-Borg J, Stricher F (2005). Prediction of water and metal binding sites and their affinities by using the Fold-X force field.. Proc Natl Acad Sci U S A.

[59] Rossato G, Ernst B, Vedani A, Smiesko M (2011). AcquaAlta: A directional approach to the solvation of ligand-protein complexes.. J Chem Inf Model.

[60] Huang N, Shoichet BK (2008). Exploiting ordered waters in molecular docking.. J Med Chem.

[61] Verdonk ML, Chessari G, Cole JC, Hartshorn MJ, Murray CW (2005). Modeling water molecules in protein-ligand docking using GOLD.. J Med Chem.

[62] Raymer ML, Sanschagrin PC, Punch WF, Venkataraman S, Goodman ED (1997). Predicting conserved water-mediated and polar ligand interactions in proteins using a K-nearest-neighbors genetic algorithm.. J Mol Biol.

[63] Kellogg GE, Semus SF, Abraham DJ (1991). HINT: a new method of empirical hydrophobic field calculation for CoMFA.. J Comput Aided Mol Des.

[64] Chen DL, Kellogg GE (2005). A computational tool to optimize ligand selectivity between two similar biomacromolecular targets.. J Comput Aided Mol Des.

[65] Amadasi A, Spyrakis F, Cozzini P, Abraham DJ, Kellogg GE (2006). Mapping the energetics of water-protein and water-ligand interactions with the “natural” HINT forcefield: predictive tools for characterizing the roles of water in biomolecules.. J Mol Biol.

[66] Amadasi A, Surface JA, Spyrakis F, Cozzini P, Mozzarelli A (2008). Robust classification of “relevant” water molecules in putative protein binding sites.. J Med Chem.

[67] Garcia-Sosa AT, Mancera RL, Dean PM (2003). WaterScore: a novel method for distinguishing between bound and displaceable water molecules in the crystal structure of the binding site of protein-ligand complexes.. J Mol Model.

[68] Barillari C, Taylor J, Viner R, Essex JW (2007). Classification of water molecules in protein binding sites.. J Am Chem Soc.

[69] Trott O, Olson AJ (2010). AutoDock Vina: improving the speed and accuracy of docking with a new scoring function, efficient optimization, and multithreading.. J Comput Chem.

[70] Hartshorn MJ, Verdonk ML, Chessari G, Brewerton SC, Mooij WT (2007). Diverse, high-quality test set for the validation of protein-ligand docking performance.. J Med Chem.

[71] van Rossum G (1995). Python tutorial, Technical report CS-R9526, Centrum voor Wikunde en Informatica (CWI).

[72] Morris GM, Huey R, Lindstrom W, Sanner MF, Belew RK (2009). AutoDock 4 and AutoDockTools 4: Automated docking with selective receptor flexibility.. J Comp Chem.

[73] Morris GM, Goodsell DS, Halliday RS, Huey R, Hart WE (1998). Automated docking using a Lamarckian genetic algorithm and an empirical binding free energy function.. J Comp Chem.

[74] Team RCD (2011). R: A language and environment for statistical computing.

[75] Akaike H (1974). A new look at the statistical model identification.. IEEE Trans Automatic Control.

[76] Kuhn LA, Swanson CA, Pique ME, Tainer JA, Getzoff ED (1995). Atomic and residue hydrophilicity in the context of folded protein structures.. Proteins: Struc Func Genet.

[77] Israelachvili J, Pashley R (1982). The hydrophobic interaction is long range, decaying exponentially with distance.. Nature.

[78] Li A-J, Nussinov R (1998). A set of van der Waals and coulombic radii of protein atoms for molecular and solvent-accessible surface calculation, packing evaluation, and docking.. Proteins: Struc Func Genet.

[79] Narten A, Levy H (1971). Liquid water: Molecular correlation functions from X-ray diffraction.. J Chem Phys.

