# Supplementary material for: Rapid and Accurate Prediction and Scoring of Water Molecules in Protein Binding Sites
Source: PLoS One. 2012 Mar 1;7(3):e32036. doi: 10.1371/journal.pone.0032036 (PMC3291545; doi:10.1371/journal.pone.0032036)
Supplement: Text S1 — Full details of the molecular dynamics procedure and the docking procedures used for specific proteins. (DOC) [file pone.0032036.s001.doc]

**Text S1.**

**Molecular dynamics procedure**

The unliganded structures of heat shock protein 90, PIM1 kinase and penicillopsin were selected from Table 1 to further validate water docking with Vina against molecular dynamics (MD) simulations. These structures were prepared and simulated using the GROMACS version 4.5.3 [1] with the OPLSAA forcefield [2]. Bond lengths and angles were constrained using the LINCS algorithm [3] and the forces were integrated using a timestep of 0.002 ps. The nearest neighbor list was updated every 10 steps. Simulations were carried out in a cubic box with periodic boundary conditions, whose walls were initially 10 Å away from the proteins. The boxes were solvated with the TIP4P [4] water model and the systems were neutralized by replacing water molecules with sodium and chlorine ions up to a concentration of 0.15 M. The temperature was kept constant at 300 K using the Noose-Hoover thermostat [5,6 ]with a time constant of 0.1 ps. Pressure was maintained at 1 atm using the Parrinello-Rahman barostat [7] with isotropic coupling and a time constant of 1 ps and compressibility of 4.5 × 10^-5^ bar^-1^. Long range electrostatics were treated using the particle mesh Ewald [8] method with a cutoff of 10 Å.

The energy of the systems were minimized using the steepest decent algorithm and was then subject to a 200 ps simulation where the heavy atoms of the protein were restrained with a spherical harmonic potential with force constant of 1000 kJ/mol/nm^2^. Finally, 10 ns unrestrained simulations were performed.

**Docking information for structures in Table 2**

The following information includes the structures used to predict the binding site water molecules as well as the alignment root-mean-squared deviations (RMSDs) of the other protein structures to the structure that was used for docking. The docking box sizes were all 15×15×15 Å^3^. All the water molecules within these volumes were used to test the sensitivity and specificity of the WaterDock method.

GluR2 ligand binding domain:

Reference Structure: 1FTM chain A

Docking center = (77.4, 26.9, 42.2)

Table S1a.

| Structure | RMSD (Å) |
| --- | --- |
| 1FTM chain B | 0.21 |
| 1FTM chain C | 0.21 |
| 1MY2 chain A | 0.13 |
| 1MY2 chain B | 0.23 |
| 1MY2 chain C | 0.20 |

Trypsin:

Reference Structure 1S0Q

Docking center = (56.2, 43.7, 17.0)

Table S1b.

| Structure | RMSD (Å) |
| --- | --- |
| 1UTQ | 0.14 |
| 1TPO | 0.2 |

HIV:

Reference Structure 2ZYE

Docking center = (14.1, 21.9, 18.0)

Table S1c.

| Structure | RMSD (Å) |
| --- | --- |
| 3FX5 | 0.29 |
| 1HPX | 0.41 |

Concavalin A:

Reference Structure 1NLS

Docking center = (68.1, 17.6, 20.6)

Table S1d.

| Structure | RMSD (Å) |
| --- | --- |
| 1QNY | 0.18 |
| 1GKB chain A | 0.21 |
| 1GKB chain B | 0.20 |
| 1JBC | 0.10 |

Gluthalione S.T.:

Reference Structure 1K3Y

Docking center = (81.3, 28.5, 14.8)

Table S1e.

| Structure | RMSD (Å) |
| --- | --- |
| 1K3Y chain B | 0.14 |
| 1K3L chain A | 0.01 |
| 1K3L chain B | 0.14 |

Ribonuclease A:

Reference Structure 1KF5

Docking center = (6.8, -20.8, -15.4)

Table S1f.

| Structure | RMSD (Å) |
| --- | --- |
| 1FS3 | 0.12 |
| 5RSA | 0.29 |

Carbonic Anhydrase II:

Reference Structure 3KS3

Docking center = (-5.7, 3.0, 13.3)

Table S1g.

| Structure | RMSD (Å) |
| --- | --- |
| 2ILI | 0.08 |
| 3MWO chain A | 0.10 |
| 3MWO chain B | 0.14 |

**References**

1. Van Der Spoel D, Lindahl E, Hess B, Groenhof G, Mark AE, et al. (2005) Gromacs: Fast, flexible, and free. J Comput Chem 26: 1701-1718.

2. Jorgensen WL, Maxwell DS, Tirado-Rives J (1996) Development and testing of the opls all-atom force field on conformational energetics and properties of organic liquids. J Am Chem Soc 118: 11225-11236.

3. Hess B, Bekker J, Berendsen HJC, Fraaije JGEM (1997) Lincs: A linear constraint solver for molecular simulations. J Comp Chem 18: 1463-1472.

4. Jorgensen WL, Chandresekhar J, Madura JD, Impey RW, Klein ML (1983) Comparison of simple potential functions for simulating liquid water. J Chem Phys 79: 926-935.

5. Hoover WG (1985) Canonical dynamics: Equilibrium phase-space distributions. Phys Rev A31: 1695-1697.

6. Nose S (1984) A molecular dynamics method for simulations in the canonical ensemble. Mol Phys 52: 255-268.

7. Parinello M, Rahman A (1981) Polymorphic transitions in single crystals - a new molecular dynamics method. J Appl Phys 52: 7182-7190.

8. Darden T, York D, Pedersen L (1993) Particle mesh ewald - an n.Log(n) method for ewald sums in large systems. J Chem Phys 98: 10089-10092.
