# Supplementary material for: Rapid and Accurate Prediction and Scoring of Water Molecules in Protein Binding Sites
Source: PLoS One. 2012 Mar 1;7(3):e32036. doi: 10.1371/journal.pone.0032036 (PMC3291545; doi:10.1371/journal.pone.0032036)
Supplement: Table S1 — The X-ray crystal structures of OppA used as the test set for the water placement method. Fourteen crystal structures of OppA were used as the WaterDock test set. This test set was chosen to match the test set used by the water prediction method Acqua Alta. The water molecules used in our study are shown in Table S4. These water molecules bridge the interaction between OppA and the ligands. The listed water molecules were used to test the true positive rate of WaterDock. As all the ligands are lysine-X-lysine tripeptides, all the water molecules around the lysine residues were used to calculate WaterDock's false positive rate. (DOC) [file pone.0032036.s002.doc]

**Table S1.**

| **PDB code*** | **Resolution (Å)** | **Ligand** |
| --- | --- | --- |
| 1JET | 1.2 | KAK |
| 1JEU | 1.25 | KEK |
| 1JEV | 1.3 | KWK |
| 1B4Z | 1.75 | KDK |
| 1B5I | 1.9 | KNK |
| 1B32 | 1.75 | KMK |
| 1B3F | 1.8 | KHK |
| 1B46 | 1.8 | KPK |
| 1B51 | 1.8 | KSK |
| 1B58 | 1.8 | KYK |
| 1B5J | 1.8 | KQK |
| 1B9J | 1.8 | KLK |
| 1QKA | 1.8 | KRK |
| 1QKB | 1.8 | KVK |
