# Supplementary material for: Rapid and Accurate Prediction and Scoring of Water Molecules in Protein Binding Sites
Source: PLoS One. 2012 Mar 1;7(3):e32036. doi: 10.1371/journal.pone.0032036 (PMC3291545; doi:10.1371/journal.pone.0032036)
Supplement: Table S2 — Water molecules used in the OppA test set. (DOC) [file pone.0032036.s003.doc]

**Table S2.**

| Structure | Number of water molecules | Water residue numbers |
| --- | --- | --- |
| 1JET | 7 | Chain A: 593, 594, 622, 846, 906, 1074. Chain B: 51. |
| 1JEU | 9 | Chain A: 534, 552, 553, 587, 608, 618, 776. Chain B: 32, 89. |
| 1JEV | 7 | Chain A: 564, 601, 697, 1043. Chain B: 22, 39, 235. |
| 1B4Z | 10 | Chain A: 540, 563, 570, 712, 716, 796. Chain B: 8, 11, 101, 455. |
| 1B5I | 7 | Chain A: 606, 658, 690, 747. Chain B: 65, 79, 82. |
| 1B32 | 7 | Chain A: 566, 581, 630, 723. Chain B: 17, 27, 123. |
| 1B3F | 7 | Chain A: 583, 621, 740. Chain B: 4, 5, 10, 145. |
| 1B46 | 6 | Chain A: 574, 600, 626, 643. Chain B: 4, 5. |
| 1B51 | 9 | Chain A: 584, 600, 612, 857, 944. Chain B: 30, 31, 81, 89. |
| 1B58 | 7 | Chain A: 574, 576, 581, 667, 692. Chain B: 16, 69. |
| 1B5J | 10 | Chain A: 945, 970, 1050, 1056,1081,1149, 1158, 1359. Chain B: 11, 98. |
| 1B9J | 6 | Chain A: 555, 596, 667, 744. Chain B: 4, 163. |
| 1QKA | 6 | Chain A: 2275, 2376,2437. Chain B: 2001, 2002, 2003. |
| 1QKB | 6 | Chain A: 2338, 2370, 2410, 2430. Chain B: 2001, 2002. |
