# Supplementary material for: Rapid and Accurate Prediction and Scoring of Water Molecules in Protein Binding Sites
Source: PLoS One. 2012 Mar 1;7(3):e32036. doi: 10.1371/journal.pone.0032036 (PMC3291545; doi:10.1371/journal.pone.0032036)
Supplement: Table S3 — Results of different water docking methods (performed on structures in Table 2 of the main manuscript). The final WaterDock method was chosen as the one that predicted the most number of consensus water molecules for the lowest false positive rate. Various docking parameters were experimented with as well as different clustering methods. To demonstrate how changing the docking or clustering parameters affects the prediction accuracy, some of the results of different water prediction methods are shown. The success rates shown are for a maximum error of 2 Å. The final method, shown in the bottom row of this table, was chosen after an exhaustive parameter and methods search. (DOC) [file pone.0032036.s004.doc]

**Table S3**

|  | Methods |  |  |  | Results |  |
| --- | --- | --- | --- | --- | --- | --- |
| Number of docking runs | Exhaustiveness | Clustering method | Clustering cutoff | Consensus waters predicted (%) | False Positives (%) | Mean Error (Å) |
| 1 | 10 | single linkage | 1.6 Å | 77 | 25 | 0.81 |
| 1 | 20 | single linkage | 1.6 Å | 78 | 22 | 0.76 |
| 3 | 10 | single linkage | 1.6 Å | 85 | 27 | 0.74 |
| 3 | 20 | single linkage | 1.6 Å | 86 | 23 | 0.78 |
| 3 | 20 | single linkage | 1.5 Å | 88 | 26 | 0.78 |
| 3 | 20 | single linkage | 1.7 Å | 83 | 23 | 0.78 |
| 3 | 20 | complete | 1 Å | 90 | 35 | 0.74 |
| 3 | 20 | complete | 1.6 Å | 90 | 28 | 0.78 |
| 3 | 20 | complete | 1.9 Å | 86 | 25 | 0.79 |
| 3 | 20 | Ward | 1 Å^2^ | 91 | 35 | 0.74 |
| 3 | 20 | Ward | 3 Å^2^ | 89 | 27 | 0.77 |
| 3 | 20 | Ward | 6 Å^2^ | 80 | 19 | 0.79 |
| **3** | **20** | **2 × single linkage** | **0.5 Å and 1.6 Å** | **87** | **19** | **0.78** |
