# Supplementary material for: Rapid and Accurate Prediction and Scoring of Water Molecules in Protein Binding Sites
Source: PLoS One. 2012 Mar 1;7(3):e32036. doi: 10.1371/journal.pone.0032036 (PMC3291545; doi:10.1371/journal.pone.0032036)
Supplement: Table S4 — Classification accuracies for conserved and displaced waters. Three water scoring energy terms were established to describe a water molecules binding energy (AutoDock Vina's hydrogen bonding term) and the water molecules' local environment with our hydrophilic and hydrophobic terms. These scores were used in 2 bagged tree classifiers that predicted whether water molecules were displaced or conserved. The probabilistic classifiers were fit using all combinations of the water scores. Cross validation results are shown and demonstrate that all three scores must be included for maximum accuracy. (DOC) [file pone.0032036.s005.doc]

**Table S4.**

| **Terms** | **Total (%)** | **Conserved (%)** | **Displaced (%)** |
| --- | --- | --- | --- |
| H-bond | 67 | 64 | 71 |
| Hydrophilicity | 66 | 68 | 64 |
| Hydrophobicity | 62 | 43 | 86 |
| H-bond and Hydrophilicity | 74 | 69 | 81 |
| H-bond and Hydrophobicity | 73 | 68 | 79 |
| Hydrophilicity and Hydrophobicity | 71 | 65 | 79 |
| **All** | **75** | **70** | **81** |
