# Supplementary material for: Rapid and Accurate Prediction and Scoring of Water Molecules in Protein Binding Sites
Source: PLoS One. 2012 Mar 1;7(3):e32036. doi: 10.1371/journal.pone.0032036 (PMC3291545; doi:10.1371/journal.pone.0032036)
Supplement: Table S5 — Classification accuracies for waters displaced by polar and non-polar groups. The probabilistic classifiers were fit using all combinations of the water scores as for Table S5. (DOC) [file pone.0032036.s006.doc]

**Table S5.**

| **Terms** | **Total (%)** | **Polar displaced (%)** | **Non-polar displaced (%)** |
| --- | --- | --- | --- |
| H-bond | 73 | 75 | 71 |
| Hydrophilicity | 75 | 70 | 79 |
| Hydrophobicity | 69 | 63 | 74 |
| H-bond and Hydrophilicity | 78 | 81 | 75 |
| H-bond and Hydrophobicity | 77 | 79 | 76 |
| Hydrophilicity and Hydrophobicity | 77 | 74 | 81 |
| **All** | **80** | **82** | **79** |
